# Folliculin mutations are not associated with severe COPD

**DOI:** 10.1186/1471-2350-9-120

**Published:** 2008-12-30

**Authors:** Michael H Cho, Barbara J Klanderman, Augusto A Litonjua, David Sparrow, Edwin K Silverman, Benjamin A Raby

**Affiliations:** 1 Channing Laboratory, Department of Medicine, Brigham and Women's Hospital; and Harvard Medical School, Boston, MA, USA; 2 Division of Pulmonary and Critical Care Medicine, Department of Medicine, Brigham and Women's Hospital; and Harvard Medical School, Boston, MA, USA; 3 Veterans Affairs Boston Healthcare System and Boston University Schools of Public Health and Medicine, Boston, MA, USA

## Abstract

**Background:**

Rare loss-of-function folliculin (*FLCN*) mutations are the genetic cause of Birt-Hogg-Dubé syndrome, a monogenic disorder characterized by spontaneous pneumothorax, fibrofolliculomas, and kidney tumors. Loss-of-function folliculin mutations have also been described in pedigrees with familial spontaneous pneumothorax. Because the majority of patients with folliculin mutations have radiographic evidence of pulmonary cysts, folliculin has been hypothesized to contribute to the development of emphysema.

To determine whether folliculin sequence variants are risk factors for severe COPD, we genotyped seven previously reported Birt-Hogg-Dubé or familial spontaneous pneumothorax associated folliculin mutations in 152 severe COPD probands participating in the Boston Early-Onset COPD Study. We performed bidirectional resequencing of all 14 folliculin exons in a subset of 41 probands and subsequently genotyped four identified variants in an independent sample of345 COPD subjects from the National Emphysema Treatment Trial (cases) and 420 male smokers with normal lung function from the Normative Aging Study (controls).

**Results:**

None of the seven previously reported Birt-Hogg-Dubé or familial spontaneous pneumothorax mutations were observed in the 152 severe, early-onset COPD probands. Exon resequencing identified 31 variants, including two non-synonymous polymorphisms and two common non-coding polymorphisms. No significant association was observed for any of these four variants with presence of COPD or emphysema-related phenotypes.

**Conclusion:**

Genetic variation in folliculin does not appear to be a major risk factor for severe COPD. These data suggest that familial spontaneous pneumothorax and COPD have distinct genetic causes, despite some overlap in radiographic characteristics.

## Background

Rare mutations in the folliculin gene (*FLCN*) have been implicated in two genetic syndromes with shared pulmonary manifestations of spontaneous pneumothorax and lung cyst formation: Birt-Hogg-Dubé syndrome (BHD, MIM 135150) and familial spontaneous pneumothorax (FSP, MIM 173600)[1]. BHD is a rare autosomal dominant monogenic disorder characterized by follicular hamartomas, renal tumors, and spontaneous pneumothorax, with an age-adjusted odds ratio of pneumothorax of 50 compared to unaffected family members[2]. FSP – defined as idiopathic spontaneous pneumothorax clustering in families in the absence of other pulmonary or systemic disease (including BHD) – is estimated to account for up to 11.5% of all cases of spontaneous pneumothorax; autosomal dominant and X-linked patterns of inheritance have been described [3,4]. Rare, loss-of-function mutations in the folliculin gene have been found in both BHD and FSP without other BHD manifestations, suggesting a shared molecular etiology; the latter cases may also represent undetected cases of BHD[5]. In total, more than 30 folliculin truncating frameshift, nonsense, or splice site mutations have been found[6-18].

BHD- and FSP-associated folliculin mutations confer distinct clinical and histopathologic pulmonary manifestations, including recurrent, idiopathic pneumothoraces (typically at a young age of onset)[3,2,7,19] and numerous parenchymal lung cysts in atypical locations (extra-apical locations as compared to predominantly apical locations observed in idiopathic spontaneous pneumothorax)[8,7,20,12]. Familial clustering of recurrent pneumothorax is common in both conditions (and defines FSP), and several radiographic surveys of asymptomatic related family members carrying folliculin mutations have confirmed a high frequency of lung cysts. For example, in one extended family with FSP, all 13 folliculin mutation carriers had cysts on chest CT, though only 5 had a history of pneumothorax[8]. Similarly, in 198 BHD subjects who underwent chest CT, 177 (89%) had one or more lung cysts, though the prevalence of pneumothorax was only 24%[19].

While these cystic changes are radiographically distinct from common forms of emphysema, increasing severity of folliculin-associated cystic changes are correlated with cigarette smoking[21]. In addition, emphysema has been reported in lung resection specimens from non-smokers with folliculin mutations and FSP[7]. Some have speculated that folliculin – perhaps by regulating processes of lung growth, or altering inflammation or matrix degradation and remodelling – may also be involved in the pathogenesis of generalized, more common forms of COPD[8,7]. We sought to test this hypothesis by characterizing the spectrum of folliculin variation in a cohort of patients with severe, early-onset COPD (nearly all with emphysema[22]) and by testing several common folliculin variants for association with lung function and emphysema in an independent case-control study.

## Results

Baseline characteristics for the cohorts are presented in Table 1. There was a notable female predominance in the Boston Early-Onset COPD Study cohort, as previously reported[23]. The EOCOPD patients had lower lung function than the NETT subjects, despite being substantially younger and reporting fewer pack-years of smoking history. Predictably, NETT subjects had significantly more pack-years of smoking and had lower lung function (p < 0.0001) than NAS subjects.

**Table 1:** Baseline characteristics of cohorts

	**EOCOPD (n = 152)**	**NETT (n = 345)**	**NAS (n = 420)**
Age, years	47.9 (4.7)	67.4 (6.0)	66.9 (8.6)

Male	41 (27.0%)	222 (64.4%)	420 (100%)

Pack-years of smoking	38.6 (20.8)	66.7 (30.9)	33.0 (22.8)

FEV_1 _(% Predicted)*	23.0 (9.8)	28.1 (7.5)	99.0 (12.0)

EOCOPD = Boston Early-Onset COPD Study; NETT = National Emphysema Treatment Trial; NAS = Normative Aging Study

Reported as mean (SD) or no. (%)

*Reference values for NETT and EOCOPD from Crapo[42], for NAS from O'Connor[43].

We first assessed whether folliculin mutations known to cause either BHD or FSP were observed among 152 EOCOPD probands. We genotyped 7 previously reported folliculin mutations, of which 2 have been observed only in FSP patients, 4 have been observed only in patients with BHD-associated pneumothorax or BHD-associated lung cysts, and 1 has been reported in both conditions (Table 2). None of these mutations were observed in any of the severe, early-onset COPD probands, suggesting that these rare variants confer distinct clinical phenotypes of BHD or FSH but do not commonly cause severe, early-onset COPD.

**Table 2:** BHD/FSP mutations genotyped in Boston early-onset COPD study probands

**Name**	**Location**	**Effect**	**Disease**	**Sequence**
c.235_238delTCGG [8]	Exon 4	Frame shift	FSP	GCAGCCCGGGGCCCAAAAAG [TCGG/-] ACATGTGCGAGGCAAGTGTC

c.630_631delAGinsC [1,9]	Exon 7	Frame shift	BHD	GCATTTCAGGTGTTTGAGGC [AG/C]AGCAGTTTGGATGCCCACAG

c.923_950dup28bp [1,9]	Exon 9	Frame shift	BHD	CTCTGAGGCTGAAGAGGAGG [AGAAAGCCCCTGTGTTGCCAGAGAGTAC/++]AGAAAGCCCCTGTGTTGCCA

c.943G>T [7]	Exon 9	p.Glu315X	FSP	AGAAAGCCCCTGTGTTGCCA [G/T]AGAGTACAGAAGGGCGGGAG

c.1278insC [1,9,21,12,11,10-13,46]	Exon 11	Frame shift	BHD	GCACGTGCAGATCCCCCCCC [-/C] ACGTGCTCTCCTCAGGTGCG

c.1278delC [1,9,21]	Exon 11	Frame shift	BHD	CGCACGTGCAGATCCCCCCC [C/-] ACGTGCTCTCCTCAGGTGCG

c.1429C>T [7,9]	Exon 12	p.Arg477X	FSP/BHD	GGAGCCCTGTAGCTGCAGAC [C/T] GAGGTGGGTGCCCCCAGGCA

FSP = Familial Spontaneous Pneumothorax; BHD = Birt-Hogg-Dubé. Positions with reference to RefSeq accession number NM_144997. Nucleotide numbering uses the A of the ATG translation initiation start site as nucleotide +1.

**Figure 1:**
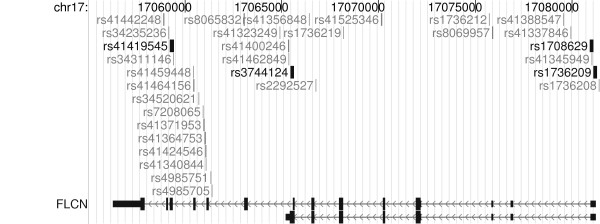
**Folliculin variants discovered by sequencing**. The folliculin gene is shown at the bottom of the figure. There are two isoforms; the longer isoform 1 (NM_144997) is on top. In the gene, thin lines represent intronic regions, with the arrowheads indicating direction of transcription. Blocks represent exons; thin blocks at either end represent the 5' and 3' untranslated regions (UTRs), while the thick blocks represent coding exons. Variants discovered by sequencing are shown above the folliculin gene, and labeled by rs number (via dbSNP) or position (referencing isoform 1) as available. Bold indicates variants subsequently genotyped in the larger cohort. UCSC Genome Browser, hg18, March 2006 Assembly.

**Figure 2:**
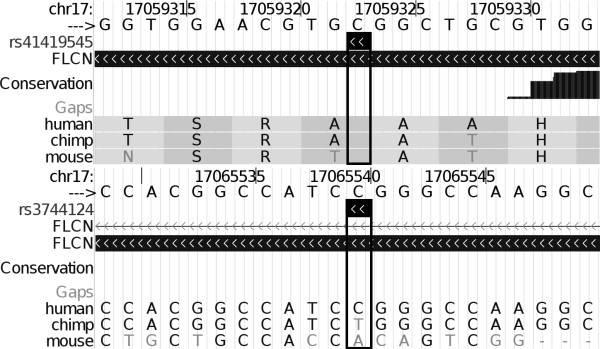
**Conservation plot of nonsynonymous SNPs in the folliculin gene by 17-way phastCons score**. (Figure 2A: rs41419545 and Figure 2B: rs3744124). The reference sequence is shown on top. The position of the variant is outlined. The thick bar below indicates that the variant is located in a coding exon. The white space below indicates low conservation across species. The bottom of each figure shows the corresponding amino acid for human, chimp, mouse, and rat, demonstrating that the amino acid at that position differs between these species. Genome Browser, hg18, March 2006 Assembly.

Although these highly penetrant mutations appear to confer distinct phenotypes of BHD and FSP but not severe COPD, it is possible that other folliculin variants could contribute to the development of EOCOPD through more subtle impact on folliculin function. To test this hypothesis, we resequenced the coding and surrounding genomic regions (13 kb total coverage) of the folliculin locus in 41 EOCOPD probands (82 chromosomes) to identify potential COPD-related variation. 31 folliculin variants were identified (Figure 1 and Table 3). Several relevant observations emerged from this effort. First, of the 31 identified variants (see Table 3) none corresponded to known BHD or FSP mutations[6-18]. Second, all 31 variants identified in the EOCOPD probands were single base-pair changes – no insertions or deletions were observed – in contrast to the spectrum of genetic variation reported in BHD and FSP which includes insertions and deletions. Third, in contrast to the deleterious effects of the variants described in BHD and FSP, none of the variants observed in the EOCOPD probands would be expected to have significant functional impact. Only two non-synonymous variants were seen: a C to T transition at c.1333 (rs41419545) in folliculin isoform 1 [GenBank:NM_144997] resulting in a substitution of alanine for threonine at codon 445 (p.Ala445Thr – observed once); and another C to T transition at c.871+36 (rs3744124), resulting in a substitution of glycine to arginine at codon 303 in folliculin isoform 2 ([GenBank:NM_144606]; p.Gly303Arg – observed four times). Neither of these variants were predicted to have significant functional impact based on PolyPhen analysis[24]. Moreover, although the coding regions of the folliculin gene are highly conserved across vertebrate species (average PhastCons score for coding exons of 0.91), both of these coding variants were situated in regions with poor local conservation – neither rs41419545 nor rs3744124 was conserved across 17 vertebral species (base-pair PhastCons scores of 0 for both, see Figure 2). Of the remaining 29 non-coding variants, 6 mapped to transcript – including 2 synonymous variants and 4 mapping to the 5' untranslated region (UTR) – 23 mapped to intronic sequence, and 1 was situated upstream of the gene. Aside from the two synonymous variants (which by definition do not alter protein structure), all of the identified variants, including those in the 5'UTR, mapped to regions of low conservation (PhastCons score ~0 for all).

**Table 3:** Summary of folliculin variants found by sequencing

**dbSNP**	Genomic position*	**mRNA position^†^**	**Location or ΔAA^†^**	**Alleles^†^**	**MAF**	**phastCons Score**
rs1736208	17081408	-	Genomic	G>A	0.25	0

**rs1736209**	**17081210**	**c.-487**	**5' UTR**	**G>C**	**0.27**	**0**

rs41345949	17081025	c.-302	5' UTR	C>T	0.06	0

**rs1708629**	**17081022**	**c.-299**	**5' UTR**	**A>G**	**0.43**	**0**

rs41337846	17079957	c.-228+994	IVS	T>C	0.14	0.0007

rs41388547	17079583	c.-228+1368	IVS	C>A	0.01	0

rs8069957	17075898	c.-90	5' UTR	T>C	0.01	0.001

rs1736212	17075733	c.-25+100	IVS	C>G	0.27	0

rs41525346	17070156	c.396+59	IVS	A>G	0.07	0

rs1736219	17068196	c.397-14	IVS	A>G	0.49	0

rs2292527	17066766	c.619-66	IVS	G>A	0.06	0

rs41356848	17066427	c.779+113	IVS	G>A	0.05	0

**rs3744124**	**17065540**	**c.871+36**	**IVS**^‡^	**C>T**	**0.05**	**0**

rs41462849	17065372	c.871+204	IVS	T>C	0.01	0

rs41400246	17065350	c.871+226	IVS	C>T	0.10	0

rs41323249	17064892	c.871+684	IVS	C>T	0.13	0

rs8065832	17063052	c.1062+6	IVS	A>G	0.48	0.008

rs4985705	17061393	c.1063-172	IVS	C>G	0.41	0

rs4985751	17061338	c.1063-117	IVS	G>A	0.02	0

rs41340844	17061077	c.1176+31	IVS	C>T	0.01	0

rs41424546	17061069	c.1176+39	IVS	C>T	0.10	0

rs41364753	17061040	c.1176+68	IVS	C>G	0.04	0

rs41371953	17060974	c.1176+134	IVS	C>G	0.04	0

rs7208065	17060929	c.1176+179	IVS	C>T	0.42	0

rs34520621	17060707	c.1177-165	IVS	G>A	0.04	0

rs41464156	17060450	c.1269	p.His423His	G>A	0.01	0.86

rs41459448	17060441	c.1278	p.Ile426Ile	G>A	0.01	0.99

rs34311146	17059414	c.1301-59	IVS	G>A	0.30	0

**rs41419545**	**17059323**	**c.1333**	**p.Ala445Thr**	**C>T**	**0.01**	**0**

rs34235236	17059167	c.1433-38	IVS	T>C	0.17	0

rs41442248	17058903	c.1538+121	IVS	G>A	0.01	0

MAF = minor allele frequency in sequenced subjects. ΔAA = change in amino acid.

Variants genotyped indicated in bold.

*Referenced to chromosome 17 on the Human Mar. 2006 (hg18) assembly

†Referenced to the positive strand and folliculin isoform 1 [GenBank:NM_144997]

^‡^p.Gly303Arg in isoform 2 [GenBank:NM_144606]

Our resequencing results suggest that COPD is not caused by rare, severely deleterious folliculin variants. However, several more common variants were identified that mapped to the folliculin transcript, raising the possibility that folliculin variants with more subtle functional impact may contribute to COPD pathogenesis. To assess this possibility, we genotyped four variants that mapped to the folliculin transcript – the two nonsynonymous variants (rs3744124 and rs41419545) and two common 5' UTR variants (rs1708629 and rs1736209) – in 345 NETT subjects and 420 NAS controls and tested for evidence of genetic association with the presence or absence of COPD. We also performed population-based association analysis of quantitative emphysema phenotypes within NETT COPD cases. All variants were in Hardy-Weinberg equilibrium in the NAS controls. In keeping with the sequence analysis above, none of these four variants was associated with COPD susceptibility or emphysema phenotypes, as evidenced by nearly identical genotype distributions for all four variants between emphysema cases and healthy smoking controls (Table 4). In addition, there was no association for any of the variants with four measures of emphysema severity and distribution in the NETT cases: radiologist-determined upper lobe emphysema predominance, radiologist-determined overall emphysema severity, quantitative difference between apical and basilar emphysema (% of lung < 950 HU) and total percentage of affected lung < -950 HU (data not shown). Additional association testing under recessive and dominant models did not change these results.

**Table 4:** Case-control association analysis of selected variants

**SNP**	**Genotype and Frequency**			**P value for Genotype**
rs41419545	CC	CT		1.0
NAS	0.99	0.01		
NETT	0.99	0.01		

rs3744124	CC	CT		0.90
NAS	0.91	0.09		
NETT	0.91	0.09		

rs1708629	AA	AG	GG	0.28
NAS	0.28	0.49	0.23	
NETT	0.34	0.45	0.22	

rs1736209	GG	CG	CC	0.24
NAS	0.52	0.39	0.10	
NETT	0.58	0.34	0.08	

## Discussion

The function of the folliculin gene is unknown. Evidence to date suggests its function as a tumor suppressor[25-28] possibly through the mTOR pathway[29,30]. While these studies help elucidate the pathways by which folliculin mutations lead to tumorigenesis, the role of folliculin in the lung is much less clear. However, high prevalence of atypical lung cyst formation among carriers of rare folliculin mutations (89%–100%), together with evidence of folliculin expression in lung macrophages, lung fibroblasts, and type I pneumocytes [26], has led to speculation that folliculin plays an important role in establishing or maintaining extracellular matrix and pulmonary parenchymal integrity. It has been hypothesized that folliculin may contribute to the development of emphysema and COPD in the general population[7,8]. We sought to address this question by characterizing the spectrum of folliculin sequence variation in a cohort of patients with severe early-onset COPD and in a case-control study of cases with severe emphysema and control smokers with normal spirometry. Using a variety of approaches including rare-variant screening, variation discovery, and genetic association testing of common variation, we found no evidence to support a role for folliculin as a genetic determinant of severe COPD, in that (1) previously reported BHD or FSP-associated folliculin mutations were not observed in any of our severe-early onset COPD probands; (2) resequencing of the folliculin locus did not reveal any protein truncating mutations; and (3) no evidence of genetic association was found between four common folliculin variants with either COPD susceptibility or emphysema distribution or severity.

Despite our comprehensive evaluation, we must emphasize the difficulty in making definitive statements regarding lack of association in genetic association studies, particularly in the context of rare sequence variants. Traditionally designed association analyses may be unable to detect association for rare variants that confer modest genetic effects due to inadequate statistical power [31,32]. In addition, rare variants may be limited to a specific ethnic group[33]; our subjects were nearly all Caucasian. While we recognize that our sample size is relatively modest, it is comparable in size to the aforementioned BHD cohorts [19]. By sampling 82 chromosomes, our detection rate for a variant of 5% frequency was > 99%[34]. Moreover, given the very high penetrance of lung cyst formation among folliculin mutation carriers, our cohort was of sufficient size to detect mutation carriers if mutations of similar effect were commonly associated with COPD. In addition, for a common variant (allele frequency of 30%) we had > 80% power to detect an odds ratio of 1.4. We also note that our cohort of severe, early-onset COPD (EOCOPD) probands represents an extreme form of the COPD spectrum and is thus more likely to be enriched for functional rare genetic variation that influences lung structure and function [35-37]. Absence of functional folliculin mutations in this cohort suggests that similar variation is not likely present in less severe forms of COPD.

Another important challenge in studies of rare genetic variation is the extent of genetic coverage. While we recognize that we did not screen for all known BHD mutations, our survey was comprehensive in that our genotyping coverage included those mutations that explain the bulk of BHD and FSP cases[9,5]. With regard to our survey of common genetic variation, though we only genotyped four variants, we note that it is unlikely that we failed to capture occult structural variation given the paucity of coding variation identified during our resequencing efforts. The folliculin locus demonstrates very strong phylogenetic conservation across mammalian species, which suggests that the folliculin locus could be under purifying selection, reducing the population prevalence of deleterious mutations in general, particularly common variation. Thus, while we cannot exclude the possibility that untested variants of very modest effect may influence COPD susceptibility, our results exclude the folliculin locus as a major determinant of severe COPD in the general population.

Though much of the attention in the study of complex traits like COPD has focused on the contribution of common sequence variation, there is increasing realization that rare sequence variants may also explain a substantial proportion of the genetic risk, particularly in subjects with extreme phenotypic manifestations[36]. In addition to the well-documented role of alpha-1 antitrypsin as a rare genetic risk factor for COPD, we have recently described a rare functional mutation in the terminal exon of the elastin gene that segregates with disease status in a family of severe early-onset COPD and was observed in 1.25% of subjects participating in the NETT Genetics Ancillary Study as compared to 0.55% in the NAS cohort[37]. Though these early studies support an important contribution of rare variants to the genetic architecture of COPD, more comprehensive surveys of large numbers of genes will be required to clarify their role. With the advent of a new generation of high-throughput sequencing platforms, such analyses will soon be feasible and will ultimately provide new insights into the pathogenesis of COPD.

## Conclusion

Folliculin has been hypothesized to play a role in emphysema, and rare folliculin mutations have been associated with cyst formation and spontaneous pneumothorax. However, genetic variation in folliculin does not appear to be a major risk factor for severe COPD. These data suggest that familial spontaneous pneumothorax and COPD have distinct genetic causes, despite some overlap in radiographic characteristics.

## Methods

Details of subject recruitment and phenotyping in the Boston Early-Onset COPD Study (EOCOPD), the National Emphysema Treatment Trial (NETT), and the Normative Aging Study (NAS) have been reported previously [38-41]. Probands in the Boston Early-Onset COPD Study had physician-diagnosed COPD, FEV_1 _< 40% predicted[42], age < 53 years, and no severe alpha-1 antitrypsin deficiency. NETT participants had physician-diagnosed COPD, FEV_1 _≤ 45% predicted[42], evidence of hyperinflation on pulmonary function testing, and bilateral emphysema on CT scan. None of the NETT subjects included in our study had severe alpha-1 antitrypsin deficiency. NAS controls were healthy men recruited through the Veterans Administration (VA) of Greater Boston[40] with at least 10 pack years of cigarette smoking, without airflow obstruction (FEV_1 _> 80% predicted[43] and FEV_1_/FVC > 90% predicted[42]). The NETT and NAS participants were all Caucasian; two of the Boston Early-Onset COPD Study probands included in this study were African-American.

Participants in the Boston Early-Onset COPD Study and the NETT Genetics Ancillary study gave written informed consent. Anonymized data were used for the NAS participants, as approved by the Partners Healthcare Human Research Committee and the IRB of the VA Hospitals. The appropriate institutional review boards approved all studies.

Seven BHD- or FSP-related folliculin mutations – including three reported in FSP, and the two most common mutations in BHD – were genotyped in 152 Boston Early-Onset COPD Study probands (Table 2). Bidirectional resequencing of the folliculin gene was performed in 41 EOCOPD probands. Selected variants were genotyped in 345 NETT subjects and 420 NAS controls. Additional details of genotyping and sequencing methods are available Additional file 1. Evolutionary conservation was assessed at single nucleotides and with 20 bp of surrounding sequence using the 17-way phastCons score[44] available through the UCSC genome browser (; March 2006 assembly).

Statistical analysis was performed in SAS 9.1 (SAS Institute, Cary, NC). Baseline characteristics in NETT versus NAS and NETT versus EOCOPD subjects were compared using Fisher's exact or Wilcoxon test, as appropriate. Hardy-Weinberg equilibrium was assessed in control subjects using an exact test. Genotype frequencies were compared using Fisher's exact test. In the NETT cohort, four CT emphysema phenotypes were analyzed. Two were based on radiologist assessments: 1) upper lobe emphysema predominance and 2) overall emphysema severity on a scale of 0 to 24. Two were quantitative scores using a cutoff of -950 Hounsfield Units: 1) the difference between apical and basilar emphysema and 2) the total percentage of affected lung. The upper lobe and apical/basilar phenotypes were chosen based on the extra-apical predominance of folliculin associated lung cysts. The quantitative densitometric phenotypes were added due to reported interobserver variability in CT phenotypes[22]. All four phenotypes were analyzed under an additive genetic model in univariate analysis and in a multivariate analysis adjusting for age, sex, post-bronchodilator FEV_1 _% predicted, and pack-years of cigarette smoking. Power calculations for discovery of novel exonic variants were performed using the exact binomial in SAS 9.1 (SAS Institute, Cary, NC), for 82 independent chromosomes. Power calculations for genotyping in NETT and NAS were performed using Quanto[45], under a log-additive model.

## Abbreviations

BHD: Birt-Hogg-Dubé Syndrome; EOCOPD: Boston Early-Onset COPD Study; FLCN: Folliculin; FSP: Familial Spontaneous Pneumothorax; MAF: Minor allele frequency; MIM: Online Mendelian Inheritance in Man; NAS: Normative Aging Study; NETT: National Emphysema Treatment Trial; UTR: Untranslated region.

## Authors' contributions

MHC performed the analysis and drafted the manuscript. BJK supervised the genotyping and sequencing. AAL and DS participated in study design and coordination and assisted in analysis. BAR and EKS conceived of the study and participated in its design and coordination. BAR assisted in analysis and helped to draft the manuscript. All authors read and approved the final manuscript.

## Pre-publication history

The pre-publication history for this paper can be accessed here:



## Supplementary Material

Additional file 1 **Supplemental methods** Click here for file
