# Supplementary material for: Folliculin mutations are not associated with severe COPD
Source: BMC Med Genet. 2008 Dec 30;9:120. doi: 10.1186/1471-2350-9-120 (PMC2636779; doi:10.1186/1471-2350-9-120)
Supplement: Additional file 1 — Supplemental methods [file 1471-2350-9-120-S1.doc]

**SUPPLEMENTAL METHODS**

Four insertion / deletion mutations (nt733delTCGG, nt1421-1448dup28bp, nt1776insC, and nt1776delC) were genotyped using fluorophore-labeled polymerase chain reactions (PCR). Genotype calls were performed by fragment length analysis on the ABI 3100 Genetic Analyzer (Applied Biosystems, Foster City, CA). The remaining mutations (nt1130delAGinsC, nt1441G>T, nt1927C>T) were genotyped using the 5’ to 3’ exonuclease TaqMan®[1] method using ABI Pre-Designed SNP Genotyping Assays as per standard protocol. Because the mutations were expected to be rare, control reactions were designed to test that the Taqman probes could indeed detect heterozygotes and homozygotes for the mutant alleles, using synthetic oligonucleotides carrying the mutant allele.

Bidirectional resequencing of the folliculin gene was performed using dye terminator dideoxy sequencing chemistry (Applied Biosystems, Foster City, CA) in 41 EOCOPD probands with larger quantities of DNA. Primers were designed to cover the exons, exon-intron junctions, and at least 50 bp of surrounding intron. 1 kbp and 2 kbp were targeted at the 5’ and 3’ ends of the gene, respectively. Sequencing reactions were run on an ABI 3730xl Genetic Analyzer, and resulting data analyzed with Phred/Phrap/Consed and Polyphred software. Selected variants were genotyped with TaqMan® assays in 345 NETT subjects and 420 NAS controls. Primer design for both sequencing and genotype assays were performed using Primer3 version 0.4.0[2](http://frodo.wi.mit.edu/cgi-bin/primer3/primer3_www.cgi).

References:

1. Holland PM, Abramson RD, Watson R, Gelfand DH: **Detection of specific polymerase chain reaction product by utilizing the 5'----3' exonuclease activity of Thermus aquaticus DNA polymerase**. *Proc Natl Acad Sci U S A* 1991, **88**(16):7276-7280.

2. Rozen S, Skaletsky H: **Primer3 on the WWW for general users and for biologist programmers**. In: *Bioinformatics Methods and Protocols: Methods in Molecular Biology.* Edited by Krawetz S, Misener S. Totowa, NJ: Humana Press; 2000: 365-386.
